# Robust Sliding Mode Control for Stochastic Uncertain Discrete Systems with Two-Channel Packet Dropouts and Time-Varying Delays

**DOI:** 10.3390/s22051965

**Published:** 2022-03-02

**Authors:** Sian Sun, Wenxia Cui, Jie Zheng

**Affiliations:** School of Mathematics, Physics and Statistics, Shanghai University of Engineering Science, Shanghai 201620, China; sunsian@163.com (S.S.); zhengjie@sues.edu.cn (J.Z.)

**Keywords:** networked control system, sliding mode control, packet dropouts, discrete delayed systems, stochastic uncertainty, sensor

## Abstract

In this paper, the control problem is investigated for discrete time-varying delayed systems with stochastic uncertainty, external disturbance, and two-channel packet dropouts. Sliding mode functions with packet loss probabilities are proposed for the packet loss problem in the sensor–controller channel and the controller–actuator channel. Furthermore, by employing the Lyapunov–Krasovskii functional, some new stability conditions are established in terms of solvable linear matrix inequalities (LMIs), and H_∞_ performance is analyzed for the sliding mode motion of the system. Meanwhile, a sliding mode controller is designed to drive the system state to the pre-designed sliding surface. Moreover, the designed controller can be robust for two-channel packet dropouts, time-varying delays, stochastic uncertainty and external disturbance. Finally, two numerical examples are given to demonstrate the feasibility of the proposed theoretical method.

## 1. Introduction

Over the last few decades, the emergence of networked control systems (NCSs) has largely solved the shortcomings of traditional control systems which are not easily scalable, inflexible, and weak against interference [1,2,3]. They have a wide range of applications in modern science, such as robot manufacturing [4], transport [5], and power transmission. However, the networked control system, which connects the original components to the communication network, inevitably introduces new problems. For example, resources are transmitted in the networked control system through the Internet. During this data transmission, the nodes will collide with each other, and competition failure or network congestion can result in packet order confusion, time delays [6,7,8], and even packet dropouts [9,10,11], due to the limitations of channel capacity or information processing speed. The sensor also has packet loss [12,13], which brings serious negative impacts that cannot be ignored. Therefore, in order to make further progress in networked control, the impact of packet dropout and time-varying delays on system stability must be reduced.

In practice, networked control systems are widely used in power systems [14], which can solve the problem of power system security control. The delay of data directly leads to the phase lag of the controlled system, affecting the dynamic performance and stability of the controlled system. Therefore, it is necessary to adopt appropriate network control technology to minimize the delay of information networks. In fact, a great deal of research has been carried out on networked control systems containing packet loss and time delays. The stochastic system approach [15] and the deterministic approach [16] are the two most commonly used methods to study the relationship between packet loss rate and NCS performance. The stochastic system approach generally uses Markov chains or Bernoulli random sequence approximations to describe the packet loss process of the system. Lu [17] proposed an improved model predictive tracking control to handle networked control systems under random packet loss and uncertainty, introducing a new state space model where tracking errors and state variables are combined and optimized, and better control performance is obtained. Shah [18] took the real-time network medium and packet loss into account, and put forward a new method for designing discrete-time sliding mode controllers using the Thiran’s delay approximation. In terms of controller, the main controllers used in the system include feedback control [19], H_∞_ control [20], predictive control [21], optimal control [22], and sliding mode control [23]. Among the above methods, the advantage of sliding mode control (SMC) is that sliding mode can be implemented completely independently of system external disturbances and parameter uptake under certain conditions. Sliding mode control is a widely used method that has a long history and has received a lot of attention [24,25,26].

The basic principle of SMC is to drive the system state to a pre-designed sliding surface and remain there for all subsequent time by correctly constructing a discontinuous control law. Therefore, a large number of methods have been provided to deal with the SMC problem for different types of systems. Zhan [27] investigated the problem of optimal tracking performance with packet loss and channel noise under channel input power constraints, exploring the conclusions related to how the packet loss probability and channel noise of the communication channel fundamentally limit the tracking capability of the control system. Niu [28] constructed discrete-time integral sliding surfaces involving fallout probabilities; the accessibility of the sliding surface was analyzed by means of the discrete-time stochastic Lyapunov method, and Niu also presented a method for estimating packet loss when generating system state information between sensors and controllers. Numerous studies have been published on sliding mode control systems with packet loss and time delays. In addition, the presence of uncertainties, external disturbances and non-linearity in the system can increase the complexity of the system analyses and modelling, especially in a system with two-channel packet dropouts. Zhang [29] investigated the sliding mode control problem for a class of discrete delayed nonlinear systems subject to randomly varying non-linearity with uncertain occurrence probabilities, and verified the effectiveness of the SMC technique. The effect of external perturbations on this system was further investigated and the feasibility was verified by Zhang [30]. However, none of the above studies have considered the effect of two-channel packets dropouts on the system. Zhang [31] presented a solution for detecting fault signals in uncertain incremental operator systems with two-channel packet dropouts and time-varying delays. Zhang [32] considered the presence of two-channel packet dropouts, uncertainty, and external disturbance in networked control systems, and proposed a novel integral sliding surface, but they did not take time delays and stochastic uncertainty into account in the system. Accordingly, it is necessary and important to consider two-channel packet dropouts, time-varying delays, external disturbances, and stochastic uncertainty in sliding mode control systems simultaneously.

Motivated by the above discussions, in this paper, a robust sliding mode controller is proposed to study the control problem of making the system stable for discrete networked control systems with two-channel packet dropouts, randomly occurring uncertainties, time delays, and external disturbance. Due to the limited bandwidth of the communication channels, sensor–controller and controller–actuator random packet dropouts may occur simultaneously in the network environment. Therefore, the system considered in this paper is more comprehensive and general. The main contributions can be summarized as follows: (1) Compared with the existing literature, the model proposed is more general in this paper. The parameter uncertainties, time-varying delays, external disturbance and two-channel packet dropouts are all considered simultaneously, which is more relevant to the actual situation. (2) Sliding surface parameters are proposed to ensure asymptotic stability and H_∞_ performance of the system in the sliding phase. (3) The sliding mode controller is designed to ensure relatively ideal system dynamics and robustness to two-channel packet dropouts, unknown parameter perturbations and external disturbances.

The arrangement of this paper is as follows. Section 2 introduces the related work on NCSs and SMC. Section 3 introduces the control system model. Section 4 proposes a sliding surface that conforms to the system. A design of the robust sliding mode controller is presented in Section 5. Numerical simulation results are shown in Section 6. Section 7 provides some conclusions.

## 2. Related Work

This section briefly reviews networked control systems and sliding mode control systems, focusing on discrete systems under the influence of many factors and the design of sliding mode controllers.

Chen [33] used a logical packet processor (DPP) to resolve the data packet disorder and data packet dropout, considered it as a special case of the time delay, and took into account time delays or data packet dropouts happening in both sensor-to-controller and controller-to-actuator channels simultaneously. However, the author did not considered the effect of uncertainty on the system and whether the system can be stable for a finite period of time. In many papers, the time delay of the system has been established as a Markov chain model. A system model has been presented for deriving channel access delay using Markov chain model [34], and Abubakar [35] used this chain model to define channel access delay in multichannel vehicular environments. Sensor-to-controller and controller-to-actuator random delays have been modeled as a Markov chain [36]; Bahreini presented a robust finite-time fault-tolerant controller for a class of uncertain NCSs with network induced random delays and actuator faults. Fault-tolerant controllers have been designed to ensure system stability. However, the external disturbance missing from this paper can also have a large impact on the system [37].

Sliding mode control (SMC) systems have been exploited to solve robust control problems of a large range of complex systems. Wang [38] presented a sliding mode dynamic output feedback controller design for Markovian jump systems (MJSs) under a communication network. The MJS model can model abrupt parameter and structural changes caused by the network. However, it cannot modeled system effects from packet loss and time delay. The SMC strategy has also been used to address the exponential stability of the switching system through an event-triggered scheme [39]; a significant advantage of event-triggered solutions is their ability to reduce redundant transmissions. However, the need for accessible system outputs have limited their practical applicability. In addition, compound controllers combined with the static output feedback of the SMC cannot eliminate the effects of external disturbances on the system [40], and conventional full-state feedback controllers [41] cannot be used when the system state cannot be fully measured.

## 3. System Description and Preliminaries

**Figure 1:**
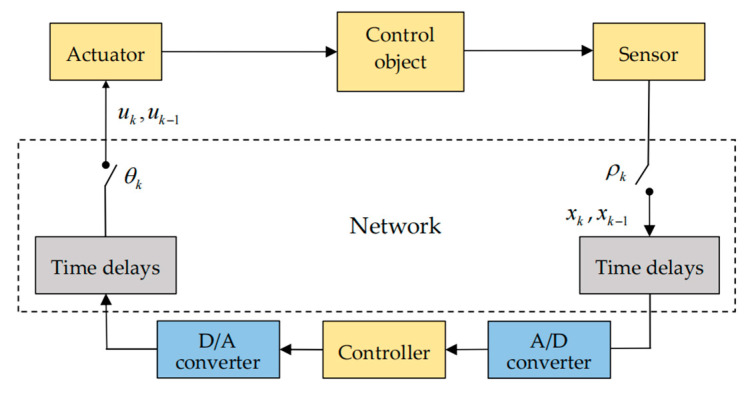
Structure of networked control system.

The structure of a class of discrete uncertain system with time-varying delays and two-channel packet dropouts is shown in Figure 1, where time delays cannot be ignored, and the breakpoints of the two channels represent the condition of packet dropouts. The sensor and actuator are time-driven, while the controllers are event-driven. For better processing of the signals, both A/D converters and D/A converters are needed before and after the controller, respectively. The control signal and feedback signal of the system are very important. These can adjust the state of the system in time and ensure the stability of the system. Here, we describe the discrete system model as follows:

(1) $$\{\begin{array}{l} x_{k+1}=(A+\alpha_{k}\Delta A)x_{k}+(A_{d}+\beta_{k}\Delta A_{d})x_{k-d_{k}}+Bu_{k}+D\omega_{k},\\ z_{k}=Cx_{k}+F\omega_{k},\\ x_{k}=\phi_{k},k=-d_{M},-d_{M}+1,\ldots ,0, \end{array}$$

where $x_{k}\in R^{n}$, $u_{k}\in R^{m}$, $z_{k}\in R^{p}$ denote the system state vector, the control input, and the control output, respectively; $\phi_{k}\in R^{q}$ is the external disturbance; $\phi_{k}$ is the state of the previous moment when the time delay occurs; $d_{k}\in [d_{m},d_{M}]$ is the time-varying delays, where $d_{m}$ and $d_{M}$ are known upper and lower bounds, respectively; $\alpha_{k}$, $\beta_{k}\in R$ are random variables, and ΔA, $\Delta A_{d}$ are mismatched system parameter perturbation. Moreover, A, $A_{d}$, B, C, D, and F are known coefficient matrices with appropriate dimensions. The matrix B is assumed to be full column rank.

In this paper, we introduce the following assumptions.

(1) The matrices ΔA and $\Delta A_{d}$ represent the mismatched norm-bounded uncertainties satisfying:

(2) $$[\begin{array}{cc} \Delta A & \Delta A_{d} \end{array}]=[\begin{array}{cc} H & H_{d} \end{array}]F_{k}N,$$

where H, $H_{d}$, and N are known matrices, $F_{k}$ is unknown matrix with $F_{k}^{T}F_{k}\leq I$.

(2) The random variable $\alpha_{k},\beta_{k}\in \{0,1\}$ is a Bernoulli white noise sequence, the probability distribution of $\alpha_{k}$, $\beta_{k}$ is $\mathrm{Pr}\{\alpha_{k}=1\}=\alpha$, $\mathrm{Pr}\{\beta_{k}=1\}=\beta$, respectively, where α,β∈[0,1].

(3) The packet dropout distribution of the two channels is assumed to obey the two-level Bernoulli random process. Denote ${\hat{x}}_{k}$ as the sensor signal that reaches the controller and ${\hat{u}}_{k}$ as the control signal that reaches the actuator. They are expressed as follows:

(3) $$\{\begin{array}{c} {\hat{x}}_{k}=(1-\rho_{k})x_{k}+\rho_{k}x_{k-1},\\ {\hat{u}}_{k}=(1-\theta_{k})u_{k}+\theta_{k}u_{k-1}, \end{array}$$

where $\rho_{k}$, $\theta_{k}\in \{0,1\}$ are parameters used to describe the packet dropout state of the two channels, when $\rho_{k}=0$ indicates that the date transmission is normal in the sensor–controller channel, and $\rho_{k}=1$ means the packet is lost. Parameter $\theta_{k}$ is the counterpart of $\rho_{k}$ in controller–actuator channel. The probability distribution of $\rho_{k},\theta_{k}$ is

(4) $$\{\begin{array}{c} \mathrm{Pr}\{\rho_{k}=1\}=\bar{\rho },\mathrm{Pr}\{\rho_{k}=0\}=1-\bar{\rho },\\ \mathrm{Pr}\{\theta_{k}=1\}=\bar{\theta },\mathrm{Pr}\{\theta_{k}=0\}=1-\bar{\theta }, \end{array}$$

where $0\leq \bar{\rho }<1,$ $0\leq \bar{\theta }<1$ are known positive constants, to denote the probability that the packet will be transmitted successfully from sensor to controller, and controller to actuator, respectively.

## 4. Design of Robust Sliding Surface and Appropriate Controller

The discrete system model studied in this paper includes these factors of time delays, two-channel packet dropouts, stochastic uncertainty, and external disturbance, to suppress the impact of packet dropouts on system stability for two channels. Similarly to Zhang et al. [32], we define the following sliding surface with packet dropout compensation function:

(5) $$s_{k}=(1-\bar{\rho })Gx_{k}+\bar{\rho }GAx_{k-2}+\bar{\theta }GBu_{k-2},$$

where G is the sliding surface parameter matrix to be designed, that GB is non-singular, we select $G=B^{T}P$ with P>0 to ensure the non-singularity of GB. It can be obtained from Equations (1) and (5) that,

(6) $$\begin{array}{cc} s_{k+1} & =(1-\bar{\rho })G(A+\alpha \Delta A)x_{k}+(1-\bar{\rho })G(A_{d}+\beta \Delta A_{d})x_{k-d_{k}}\\ & +(1-\bar{\rho })GBu_{k}+(1-\bar{\rho })GD\omega_{k}+\bar{\rho }GAx_{k-1}+\bar{\theta }GBu_{k-1}. \end{array}$$

Note that the ideal quasi-sliding mode satisfying

(7) $$s_{k+1}=s_{k}=0.$$

Then, the equivalent controller can be derived from Equations (6) and (7) as follows:

(8) $$\begin{array}{cc} u_{eq} & =-{\left(GB\right)}^{-1}[G(A+\alpha \Delta A)x_{k}+G(A_{d}+\beta \Delta A_{d})x_{k-d_{k}}+GD\omega_{k}+\lambda_{1}GAx_{k-1}]\\ & -\lambda_{2}u_{k-1}, \end{array}$$

where $\lambda_{1}=\frac{\bar{\rho }}{1-\bar{\rho }},$ $\lambda_{2}=\frac{\bar{\theta }}{1-\bar{\rho }}.$ Substituting Equation (8) into (1), the sliding mode dynamics equation is obtained as

(9) $$\begin{array}{c} x_{k+1}=(A+\alpha \Delta A-\Delta \bar{A})x_{k}+(A_{d}+\beta \Delta A_{d}-\Delta {\bar{A}}_{d})x_{k-d_{k}}-\lambda_{1}\bar{A}x_{k-1}\\ -\lambda_{2}Bu_{k-1}+\bar{D}\omega_{k}+(\alpha_{k}-\alpha )\Delta Ax_{k}+(\beta_{k}-\beta )\Delta A_{d}x_{k-d_{k}}, \end{array}$$

where $\Delta \bar{A}=B{\left(GB\right)}^{-1}G(A+\alpha \Delta A),\Delta {\bar{A}}_{d}=B{\left(GB\right)}^{-1}G(A_{d}+\beta \Delta A_{d}),\bar{A}=B{\left(GB\right)}^{-1}GA,$ $\bar{D}=$$D-B{\left(GB\right)}^{-1}GD$.

**Definition** **1.** *The system (9) is said to be admissible with an H_∞_-norm bound γ, if the system with $\omega_{k}=0$ is admissible and under the zero-initial conditions the output $z_{k}$ satisfies* $\sum_{k=0}^{\infty }E\{{\left\|z_{k}\right\|}^{2}\}\leq \gamma^{2}\sum_{k=0}^{\infty }{\left\|\omega_{k}\right\|}^{2}$.

**Lemma** **1 ** **(**[42]**).***For any real vector a, b, and matrix P>0 of appropriate dimensions, we have*

(10) $$a^{T}b+b^{T}a\leq a^{T}Pa+b^{T}Pb.$$

**Lemma** **2**  **(**[43]**).***Give appropriate dimension constant matrices $S_{1}$, $S_{2}$, $S_{3}$, where $S_{1}=S_{1}^{T}$ and $0<S_{2}=S_{2}^{T}$, then $S_{1}+S_{3}^{T}S_{2}^{-1}S_{3}<0$ if and only if*

(11) $$\left[\begin{array}{cc} S_{1} & S_{3}^{T}\\ {}* & -S_{2} \end{array}\right]<0\;\mathrm{or}\;\left[\begin{array}{cc} -S_{2} & S_{3}\\ {}* & S_{1} \end{array}\right]<0.$$

**Lemma** **3**  **(**[44]**).***Q is the real symmetric matrix, let $Q=Q^{T}$, M, and N be real matrices of compatible dimension. Then, $Q+HFN+N^{T}F^{T}H^{T}<0$ for all F satisfying $F^{T}F\leq I$, if and only if there exists a constant ε>0 such that $Q+\varepsilon HH^{T}+\varepsilon^{-1}N^{T}N<0$ or, equivalently,*

(12) $$\left[\begin{array}{ccc} Q & \varepsilon H & N^{T}\\ \varepsilon H^{T} & -\varepsilon I & 0\\ N & 0 & -\varepsilon I \end{array}\right]<0.$$

**Theorem** **1.** *The sliding mode dynamics (9) with $\omega_{k}=0$ is robustly asymptotically stable in mean square sense if there exist matrices P>0,Q>0,R>0,Λ>0, and ε>0 satisfying*

(13) $$\Psi =\left[\begin{array}{cccc} \Psi_{11} & \Psi_{12} & \Psi_{13} & \Psi_{14}\\ {}* & \Psi_{22} & 0 & \Psi_{24}\\ {}* & * & \Psi_{33} & \Psi_{34}\\ {}* & * & * & \Psi_{44} \end{array}\right]<0,$$

*where*

$$\Psi_{11}={\tilde{\prod }}_{11},\;\Psi_{12}={\tilde{\prod }}_{12},\;\Psi_{13}={\tilde{\prod }}_{13},\;\Psi_{22}=\prod_{22},\;\Psi_{33}=\prod_{33},\bar{\Lambda }={\left[{\left(GB\right)}^{-1}\right]}^{T}\Lambda {\left(GB\right)}^{-1},$$

$${\tilde{\prod }}_{11}=\left[\begin{array}{ccccc} -P+R+(d_{M}-d_{m}+1)Q & 0 & 0 & \sqrt{2\xi }A^{T}P & \sqrt{2\xi }A^{T}PB\\ {}* & -Q & 0 & 0 & 0\\ {}* & * & -R & 0 & 0\\ {}* & * & * & -P & 0\\ {}* & * & * & * & -B^{T}PB \end{array}\right],$$

$${\tilde{\prod }}_{13}=\left[\begin{array}{ccccc} 0 & 0 & 0 & 0 & 0\\ \sqrt{\xi }A_{d}{}^{T}PB & 0 & 0 & 0 & 0\\ 0 & \sqrt{g}A^{T}PB & \sqrt{g}A^{T}PB & 0 & 0\\ 0 & 0 & 0 & 0 & 0\\ 0 & 0 & 0 & \sqrt{h}B^{T}P & \sqrt{1-h}B^{T}PB \end{array}\right],{\tilde{\prod }}_{12}=\left[\begin{array}{c} \prod_{1}\\ \prod_{2}\\ 0_{3\times 5} \end{array}\right],\;\Psi_{14}=\left[\begin{array}{cccc} 0 & 0 & \varepsilon N^{T} & 0\\ 0 & 0 & 0 & \varepsilon N^{T}\\ 0 & 0 & 0 & 0\\ \sqrt{2\xi }\alpha PH & 0 & 0 & 0\\ \sqrt{2\xi }\alpha B^{T}PH & 0 & 0 & 0 \end{array}\right],\Psi_{24}=\left[\begin{array}{cccc} \sqrt{\bar{\alpha }}PH & 0 & 0 & 0\\ \sqrt{\xi }\alpha B^{T}PH & 0 & 0 & 0\\ 0 & \sqrt{2\xi }\beta PH_{d} & 0 & 0\\ 0 & \sqrt{2\xi }\beta B^{T}PH_{d} & 0 & 0\\ 0 & \sqrt{\bar{\beta }}PH_{d} & 0 & 0 \end{array}\right],$$

$$\Psi_{34}=\left[\begin{array}{c} \Psi_{34}^{*}\\ 0_{4\times 4} \end{array}\right],\Psi_{34}^{*}=[\begin{array}{cccc} 0 & \sqrt{\xi }\beta B^{T}PH_{d} & 0 & 0 \end{array}],\;\Psi_{44}=diag\{-\varepsilon I,-\varepsilon I,-\varepsilon I,-\varepsilon I\},$$

$$\prod_{1}=[\begin{array}{ccccc} 0 & \sqrt{\xi }A^{T}PB & 0 & 0 & 0 \end{array}],\;\prod_{2}=[\begin{array}{ccccc} 0 & 0 & \sqrt{2\xi }A_{d}{}^{T}P & \sqrt{2\xi }A_{d}{}^{T}PB & 0 \end{array}],$$

$$\prod_{22}=diag\{-P,-{\bar{\Lambda }}^{-1},-P,-B^{T}PB,-P\},\bar{\alpha }=\alpha (1-\alpha ),\bar{\beta }=\beta (1-\beta )$$

$$\prod_{33}=diag\{-{\bar{\Lambda }}^{-1},-B^{T}PB,-{\bar{\Lambda }}^{-1},-P,-{\bar{\Lambda }}^{-1}\},\xi =2+\lambda_{1}+\lambda_{2},$$

$$g=\lambda_{1}{}^{2}+2\lambda_{1}+\lambda_{1}\lambda_{2},h=\lambda_{2}{}^{2}+2\lambda_{2}+\lambda_{1}\lambda_{2}.$$

**Proof** **of** **Theorem** **1.** Please see Appendix A. □

**Remark** **1.** *In matrices $\bar{\Lambda }={\left[{\left(GB\right)}^{-1}\right]}^{T}\Lambda {\left(GB\right)}^{-1}$ and $G=B^{T}P$, Λ and G are unknown, which leads to the matrix inequality being non-linearly unsolvable. Therefore, it is not possible to directly calculate Λ and G, respectively, but $\bar{\Lambda }$ and P can be obtained by Equation (13), and then Λ can be calculated by $\bar{\Lambda }={\left[{\left(GB\right)}^{-1}\right]}^{T}\Lambda {\left(GB\right)}^{-1}$.*

The following theorem further analyzes the admissibility of system (9) with an H_∞_-norm bound.

**Theorem** **2.** *For a given scalar γ>0, the sliding mode dynamics (9) is robustly mean-square asymptotically stable, if there exist matrices P>0,Q>0,R>0,Λ>0, and scalar ε>0 satisfying*

(14) $$\Xi =\left[\begin{array}{cccc} \Xi_{11} & \Xi_{12} & \Xi_{13} & \Xi_{14}\\ {}* & \Xi_{22} & 0 & \Xi_{24}\\ {}* & * & \Xi_{33} & 0\\ {}* & * & * & \Xi_{44} \end{array}\right]<0,$$

*where*

$$\Xi_{11}=\left[\begin{array}{cccccc} -P+R+(d_{M}-d_{m}+1) & 0 & 0 & C^{T}F & \sqrt{2\bar{\xi }}A^{T}P & \sqrt{2\bar{\xi }}A^{T}PB\\ {}* & -Q & 0 & 0 & 0 & 0\\ {}* & * & -R & 0 & 0 & 0\\ {}* & * & * & -\gamma^{2}Ι+F^{T}F & 0 & 0\\ {}* & * & * & * & -P & 0\\ {}* & * & * & * & * & -B^{T}PB \end{array}\right],$$

$$\Xi_{12}=\left[\begin{array}{c} {\hat{\Xi }}_{12}\\ 0_{4\times 6} \end{array}\right],{\hat{\Xi }}_{12}=\left[\begin{array}{cccccc} 0 & \sqrt{\bar{\xi }}A^{T}PB & 0 & 0 & 0 & 0\\ 0 & 0 & \sqrt{2\bar{\xi }}A_{d}{}^{T}P & \sqrt{2\bar{\xi }}A_{d}{}^{T}PB & 0 & \sqrt{\bar{\xi }}A_{d}{}^{T}PB \end{array}\right],\;$$

$$\Xi_{13}=\left[\begin{array}{c} 0_{2\times 7}\\ {\bar{\Xi }}_{13}\\ 0_{1\times 7}\\ {\hat{\Xi }}_{13} \end{array}\right],{\hat{\Xi }}_{13}=[\begin{array}{ccccccc} 0 & 0 & 0 & 0 & 0 & \sqrt{\bar{h}}B^{T}P & \sqrt{1-\bar{h}}B^{T}PB \end{array}],\;$$

$${\bar{\Xi }}_{13}=\left[\begin{array}{ccccccc} \sqrt{\bar{g}}A^{T}PB & \sqrt{\bar{g}}A^{T}PB & 0 & 0 & 0 & 0 & 0\\ 0 & 0 & \sqrt{2\bar{\xi }}D^{T}P & \sqrt{2\bar{\xi }}D^{T}PB & \sqrt{\bar{\xi }}D^{T}PB & 0 & 0 \end{array}\right],\;$$

$$\Xi_{14}=\left[\begin{array}{cccc} 0 & 0 & \varepsilon N^{T} & 0\\ 0 & 0 & 0 & \varepsilon N^{T}\\ 0 & 0 & 0 & 0\\ 0 & 0 & 0 & 0\\ \sqrt{2\bar{\xi }}\alpha PH & 0 & 0 & 0\\ \sqrt{2\bar{\xi }}\alpha B^{T}PH & 0 & 0 & 0 \end{array}\right],\;$$

$$\Xi_{24}=\left[\begin{array}{cccc} \sqrt{\bar{\alpha }}PH & 0 & 0 & 0\\ \sqrt{\bar{\xi }}\alpha B^{T}PH & 0 & 0 & 0\\ 0 & \sqrt{2\bar{\xi }}\beta PH_{d} & 0 & 0\\ 0 & \sqrt{2\bar{\xi }}\beta B^{T}PH_{d} & 0 & 0\\ 0 & \sqrt{\bar{\beta }}PH_{d} & 0 & 0\\ 0 & \sqrt{\bar{\xi }}\beta B^{T}PH_{d} & 0 & 0 \end{array}\right],\;$$

$$\Xi_{22}=diag\{-P,-{\bar{\Lambda }}^{-1},-P,-B^{T}PB,-P,-{\bar{\Lambda }}^{-1}\},\Xi_{44}=diag\{-\varepsilon Ι,-\varepsilon Ι,-\varepsilon Ι,-\varepsilon Ι\},\;$$

$$\Xi_{33}=diag\{-{\bar{\Lambda }}^{-1},-B^{T}PB,-P,-B^{T}PB,-{\bar{\Lambda }}^{-1},-P,-{\bar{\Lambda }}^{-1}\},\;$$

$$\bar{\xi }=3+\lambda_{1}+\lambda_{2}\bar{g}=\lambda_{1}{}^{2}+3\lambda_{1}+\lambda_{1}\lambda_{2}\bar{h}=\lambda_{2}{}^{2}+3\lambda_{2}+\lambda_{1}\lambda_{2}.\;$$

**Proof** **of** **Theorem** **2.** Please see Appendix B. □

**Corollary** **1.** *It should be noted that the result is shown as a feasible solution for the system stability problem in the presence of two-channel packet dropouts, time delays, stochastic uncertainty and disturbances in Theorem 2. To illustrate the system’s performance with the same packet loss rates and H_∞_ performance index γ, then the feasibility problem inequality (14) can be converted into the minimization problem:*

(15) $$\min\gamma^{2}\;s.t.\mathrm{LMI}(14)$$

## 5. Design of Sliding Mode Controller

Based on the definition of the discrete arrival conditions presented in [30], our goal is to synthesize the desired sliding mode controller. The necessary performance requirements are guaranteed if the following reach conditions hold

(16) $$\{\begin{array}{c} \Delta s_{k}=s_{k+1}-s_{k}\leq -TU\mathrm{sgn}[s_{k}]-TVs_{k},\mathrm{if}\;s_{k}>0\\ \Delta s_{k}=s_{k+1}-s_{k}\geq -TU\mathrm{sgn}[s_{k}]-TVs_{k},\mathrm{if}\;s_{k}<0 \end{array}$$

where T is the sampling period, $U=\mathrm{diag}\{\mu_{1},\mu_{2},\ldots \mu_{l}\}$ and $V=\mathrm{diag}\{\nu_{1},\nu_{2},\ldots \nu_{l}\}$, where $\mu_{i}>0$ and $\nu_{i}>0$ are properly chosen scalars satisfying $0<1-Tv_{i}<1$ (i=1,2, …,l).

Uncertain terms $(1-\bar{\rho })\alpha_{k}G\Delta Ax_{k}$, $(1-\bar{\rho })\beta_{k}G\Delta A_{d}x_{k-k_{d}}$, and $(1-\bar{\rho })GD\omega_{k}$ exist in (6). Compensation must be made during controller design in order to achieve relatively desirable reach-phase performance. Then, a sliding mode controller is proposed based on the uncertainty upper bound. Set $\Delta_{a}(k)=(1-\bar{\rho })\alpha_{k}G\Delta Ax_{k},$ $\Delta_{d}(k)=(1-\bar{\rho })G$ $\cdot (A_{d}+\beta_{k}\Delta A_{d})x_{k-d_{k}}$, and $\Delta_{\omega }(k)=(1-\bar{\rho })GD\omega_{k}$ which are assumed to be bounded. We suppose that there exist known bounds ${\underline{\delta }}_{a}^{i},{\bar{\delta }}_{a}^{i},{\underline{\delta }}_{d}^{i},{\bar{\delta }}_{d}^{i},{\underline{\delta }}_{\omega }^{i}$, and ${\bar{\delta }}_{\omega }^{i}(i=1,2,\ldots ,l)$ satisfying ${\underline{\delta }}_{a}^{i}<\delta_{a}^{i}(k)<{\bar{\delta }}_{a}^{i},$ ${\underline{\delta }}_{d}^{i}<\delta_{d}^{i}(k)<{\bar{\delta }}_{d}^{i},$ ${\underline{\delta }}_{\omega }^{i}<\delta_{\omega }^{i}(k)<{\bar{\delta }}_{\omega }^{i}$, where $\delta_{a}^{i}(k),\delta_{d}^{i}(k)$, and $\delta_{\omega }^{i}(k)(i=1,2,\ldots ,l)$ are the ith element in $\Delta_{a}(k),$ $\Delta_{d}(k)$, and $\Delta_{\omega }(k),$ respectively. Define

$$\begin{array}{cc} {\hat{\Delta }}_{a}={\left[\begin{array}{cccc} {\hat{\delta }}_{a}^{1} & {\hat{\delta }}_{a}^{2} & \ldots & {\hat{\delta }}_{a}^{l} \end{array}\right]}^{T}, & {\hat{\delta }}_{a}^{i}=\frac{{\bar{\delta }}_{a}^{i}+{\underline{\delta }}_{a}^{i}}{2},\\ {\hat{\Delta }}_{d}={\left[\begin{array}{cccc} {\hat{\delta }}_{d}^{1} & {\hat{\delta }}_{d}^{2} & \ldots & {\hat{\delta }}_{d}^{l} \end{array}\right]}^{T}, & {\hat{\delta }}_{d}^{i}=\frac{{\bar{\delta }}_{d}^{i}+{\underline{\delta }}_{d}^{i}}{2},\\ {\hat{\Delta }}_{\omega }={\left[\begin{array}{cccc} {\hat{\delta }}_{\omega }^{1} & {\hat{\delta }}_{\omega }^{2} & \ldots & {\hat{\delta }}_{\omega }^{l} \end{array}\right]}^{T}, & {\hat{\delta }}_{\omega }^{i}=\frac{{\bar{\delta }}_{\omega }^{i}+{\underline{\delta }}_{\omega }^{i}}{2},\\ {\tilde{\Delta }}_{a}=\mathrm{daig}\{{\tilde{\delta }}_{a}^{1},{\tilde{\delta }}_{a}^{2},\ldots ,{\tilde{\delta }}_{a}^{l}\}, & {\tilde{\delta }}_{a}^{i}=\frac{{\bar{\delta }}_{a}^{i}-{\underline{\delta }}_{a}^{i}}{2},\\ {\tilde{\Delta }}_{d}=\mathrm{daig}\{{\tilde{\delta }}_{d}^{1},{\tilde{\delta }}_{d}^{2},\ldots ,{\tilde{\delta }}_{d}^{l}\}, & {\tilde{\delta }}_{d}^{i}=\frac{{\bar{\delta }}_{d}^{i}-{\underline{\delta }}_{d}^{i}}{2},\\ {\tilde{\Delta }}_{\omega }=\mathrm{daig}\{{\tilde{\delta }}_{\omega }^{1},{\tilde{\delta }}_{\omega }^{2},\ldots ,{\tilde{\delta }}_{\omega }^{l}\}, & {\tilde{\delta }}_{\omega }^{i}=\frac{{\bar{\delta }}_{\omega }^{i}-{\underline{\delta }}_{\omega }^{i}}{2}. \end{array}$$

Then, we can construct a discrete-time robust sliding mode controller and ensure its accessibility.

**Theorem** **3.** *For system (1) and the sliding mode function (5), the sliding mode control law (17) is chosen so that the system can reach the sliding mode surface (6):*

(17) $$\begin{array}{cc} u_{k} & =-\frac{1}{1-\bar{\rho }}{\left(GB\right)}^{-1}[TU\mathrm{sgn}[s_{k}]+(TV-Ι)s_{k}+GA{\hat{x}}_{k}+\bar{\theta }GBu_{k-1}\\ & +({\hat{\Delta }}_{a}+{\hat{\Delta }}_{d}+{\hat{\Delta }}_{\omega })+({\tilde{\Delta }}_{a}+{\tilde{\Delta }}_{d}+{\tilde{\Delta }}_{\omega })\mathrm{sgn}[s_{k}]]. \end{array}$$

**Proof** **of** **Theorem** **3.** By substituting (17) into (6), we can obtain

(18) $$\begin{array}{cc} \Delta s_{k} & =(1-\bar{\rho })G(A+\alpha \Delta A)x_{k}+(1-\bar{\rho })G(A_{d}+\beta \Delta A_{d})x_{k-d_{k}}\\ & +(1-\bar{\rho })GBu_{k}+(1-\bar{\rho })GD\omega_{k}+\bar{\rho }GAx_{k-1}+\bar{\theta }GBu_{k-1}-s_{k}\\ & =-TU\mathrm{sgn}[s_{k}]-TVs_{k}+\Delta_{a}(k)+\Delta_{d}(k)+\Delta_{\omega }(k)\\ & -({\hat{\Delta }}_{a}+{\hat{\Delta }}_{d}+{\hat{\Delta }}_{\omega })-({\tilde{\Delta }}_{a}+{\tilde{\Delta }}_{d}+{\tilde{\Delta }}_{\omega })\mathrm{sgn}[s_{k}]. \end{array}$$

It is easy to verify that the convergence law condition (16) is satisfied, so that the sliding surface (5) is accessible, and the theorem is proved. □

## 6. Numerical Example

In this section, we will give examples which drive the effectiveness of the results obtained in the previous sections.

**Example** **1.** *Consider the following uncertain system in the form of Equation (1):*

$$A=\left[\begin{array}{ccc} 0.01 & -0.01 & 0\\ 0 & 0.012 & 0.03\\ 0.003 & 0 & -0.005 \end{array}\right],A_{d}=\left[\begin{array}{ccc} 0.007 & 0 & -0.04\\ 0.02 & 0.01 & 0\\ 0.02 & 0.04 & -0.05 \end{array}\right],B=\left[\begin{array}{cc} 0.01 & 0.04\\ 0.01 & 0.12\\ 0.04 & 0.01 \end{array}\right],$$

$$C=\left[\begin{array}{ccc} 0.02 & 0 & -0.01\\ 0.01 & 0.15 & 0 \end{array}\right],D=\left[\begin{array}{c} 0.03\\ -0.02\\ 0.003 \end{array}\right],F=\left[\begin{array}{c} -0.01\\ 0.1 \end{array}\right].$$

*The parameters for packet dropouts, parameter perturbation, time-varying delays, and external perturbation are described as*

$$H^{T}=[\begin{array}{ccc} 0.4 & 0.02 & 0.1 \end{array}],H_{d}^{T}=[\begin{array}{ccc} 0.01 & -0.1 & 0.1 \end{array}],N=[\begin{array}{ccc} 0.01 & -0.5 & -0.1 \end{array}],$$

$$\alpha =0.75,\beta =0.78,\bar{\rho }=0.2,\bar{\theta }=0.2,d_{m}=2,d_{M}=5,\gamma =0.12,\omega_{k}=e^{-k}\cos k,$$

$$F_{k}=0.2\sin(k).$$

*Let* $T=0.01,\mu_{i}=v_{i}=1$ *(* i=1,2 *), and*

$${\underline{\delta }}_{a}^{i}=-\left\|GH\right\|\cdot \left\|Nx_{k}\right\|,{\bar{\delta }}_{a}^{i}=\left\|GH\right\|\cdot \left\|Nx_{k}\right\|,{\underline{\delta }}_{\omega }^{i}=-1,{\bar{\delta }}_{\omega }^{i}=1,$$

$${\underline{\delta }}_{d}^{i}=-\left\|GA_{d}x_{k-dk}\right\|-\left\|GH_{d}\right\|\cdot \left\|Nx_{k-d_{k}}\right\|,{\bar{\delta }}_{d}^{i}=\left\|GA_{d}x_{k-d_{k}}\right\|+\left\|GH_{d}\right\|\cdot \left\|Nx_{k-d_{k}}\right\|.$$

*Then, the sliding surface parameter matrix can be obtained by solving LMI (28), that is*

$$P=\left[\begin{array}{ccc} 0.1192 & 0.0092 & -0.1007\\ 0.0092 & 0.5202 & -0.0187\\ -0.1007 & -0.0187 & 0.4457 \end{array}\right],Q=\left[\begin{array}{ccc} 0.0163 & -0.0047 & -0.0130\\ -0.0047 & 0.0527 & -0.0112\\ -0.0130 & -0.0112 & 0.0454 \end{array}\right],$$

$$R=\left[\begin{array}{ccc} 0.0237 & 0.0137 & -0.0257\\ 0.0137 & 0.0195 & -0.0046\\ -0.0257 & -0.0046 & 0.0942 \end{array}\right],\Lambda =\left[\begin{array}{cc} 0.0063 & 0.0321\\ 0.0321 & 0.2909 \end{array}\right],\varepsilon =0.1319.$$

*Then, we can obtain* $G=B^{T}P=\left[\begin{array}{ccc} -0.0027 & 0.0045 & 0.0166\\ 0.0049 & 0.0626 & -0.0018 \end{array}\right].$

**Figure 2:**
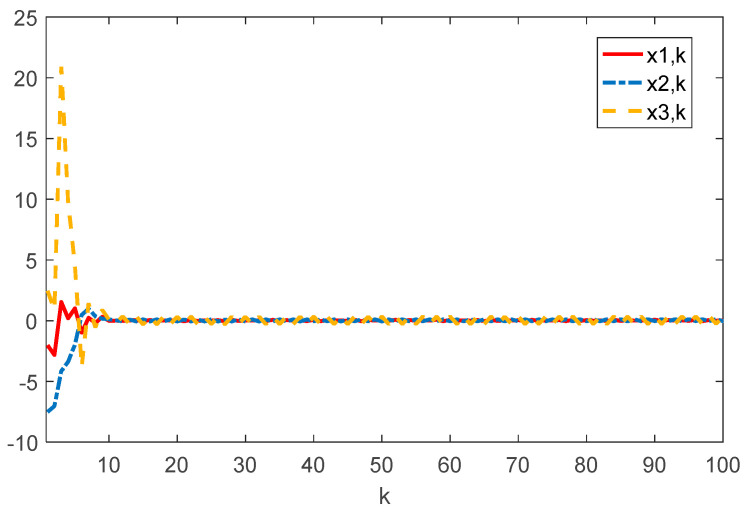
Time evolution of $x_{i\cdot k}(i=1,2,3)$ with controller (17).

**Figure 3:**
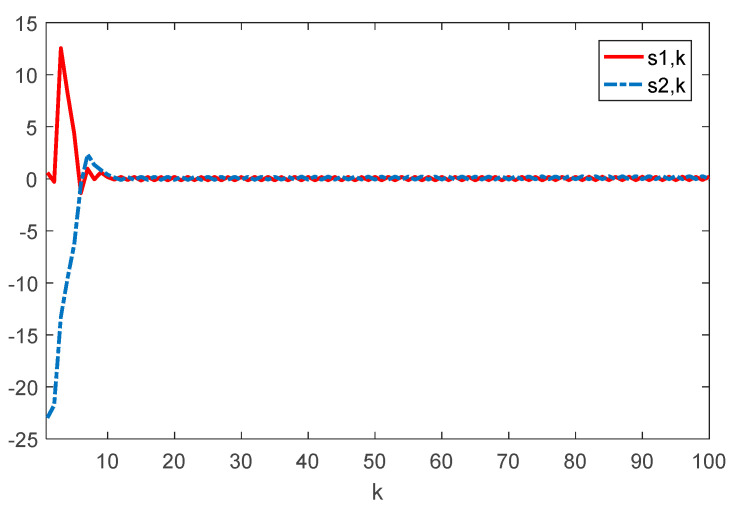
Time evolution of $s_{i\cdot k}(i=1,2)$ with controller (17).

**Figure 4:**
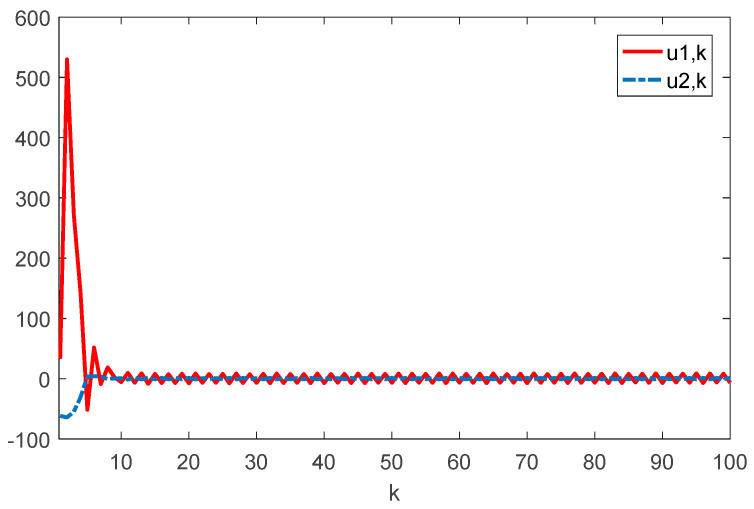
Time evolution of $u_{i\cdot k}(i=1,2)$ with controller (17).

**Figure 5:**
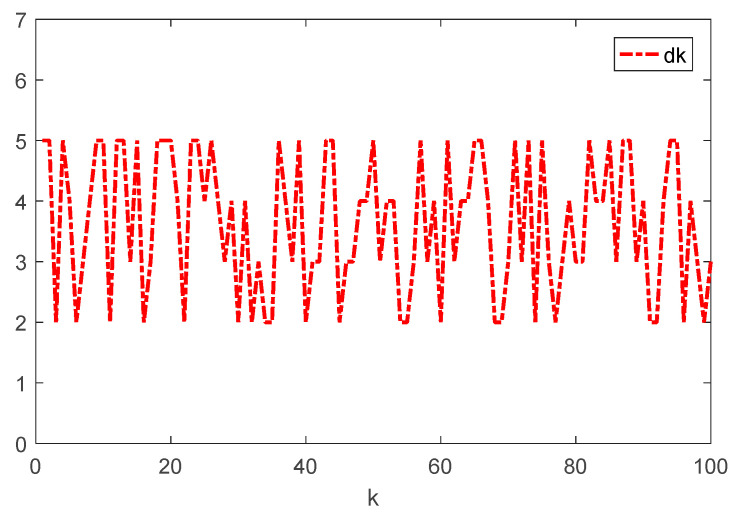
Time evolution of $d_{k}$.

Given the initial conditions, the simulation results can be given in Figure 1, Figure 2, Figure 3 and Figure 4. Among them, Figure 2 shows the state responses of the system where $x_{1,k}$, $x_{2,k}$, and $x_{3,k}$ converge to a small neighborhood of zero quickly, indicating that the considered system is H_∞_ admissible. The sliding mode function $s_{k}$ and control signal $u_{k}$ are presented in Figure 3 and Figure 4, respectively. Figure 5 shows the time delays $d_{k}$ varying with time. From the following simulations, we can conclude that the control scheme is effective.

**Example** **2.** *The coefficient matrix of the system is the same as that in Example 1. The difference is $\bar{\rho }=0.4,$ then sliding surface parameter matrix can be obtained by solving LMI (28), that is*

$$P=\left[\begin{array}{ccc} 0.1016 & -0.0038 & -0.0947\\ -0.0038 & 0.4800 & -0.0149\\ -0.0947 & -0.0149 & 0.4090 \end{array}\right],Q=\left[\begin{array}{ccc} 0.0143 & -0.0054 & -0.0119\\ -0.0054 & 0.0476 & -0.0109\\ -0.0119 & -0.0109 & 0.0405 \end{array}\right],\;$$

$$R=\left[\begin{array}{ccc} 0.0190 & 0.0084 & -0.0240\\ 0.0084 & 0.0130 & -0.0057\\ -0.0240 & -0.0057 & 0.0719 \end{array}\right],\Lambda =\left[\begin{array}{cc} 0.0038 & 0.0231\\ 0.0231 & 0.2747 \end{array}\right],\varepsilon =0.1220.$$

*Then, we can obtain $G=B^{T}P=\left[\begin{array}{ccc} -0.0028 & 0.0042 & 0.0153\\ 0.0027 & 0.0573 & -0.0015 \end{array}\right].$*

**Figure 6:**
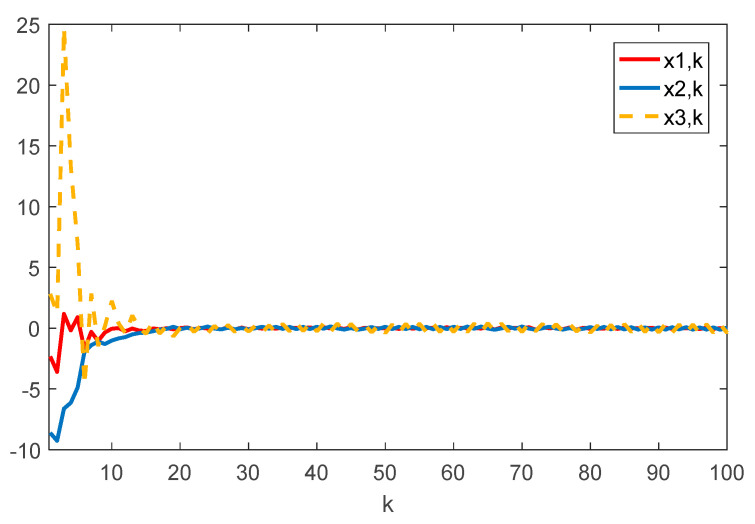
Time evolution of $x_{i\cdot k}(i=1,2,3)$ with controller (17).

**Figure 7:**
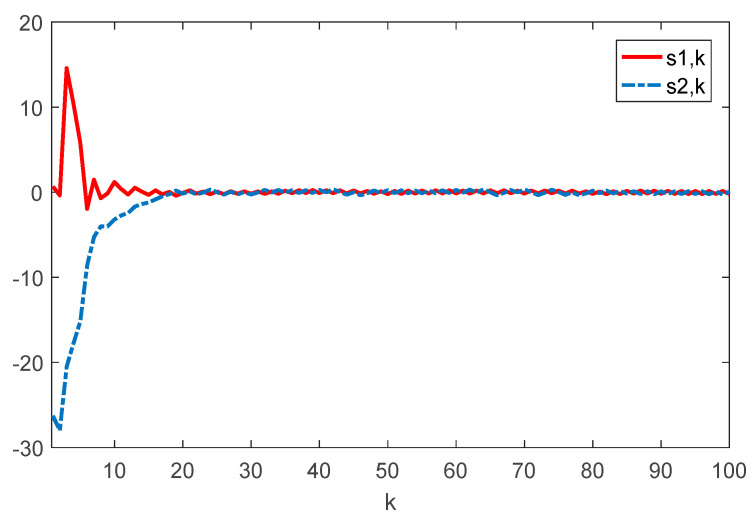
Time evolution of $s_{i\cdot k}(i=1,2)$ with controller (17).

**Figure 8:**
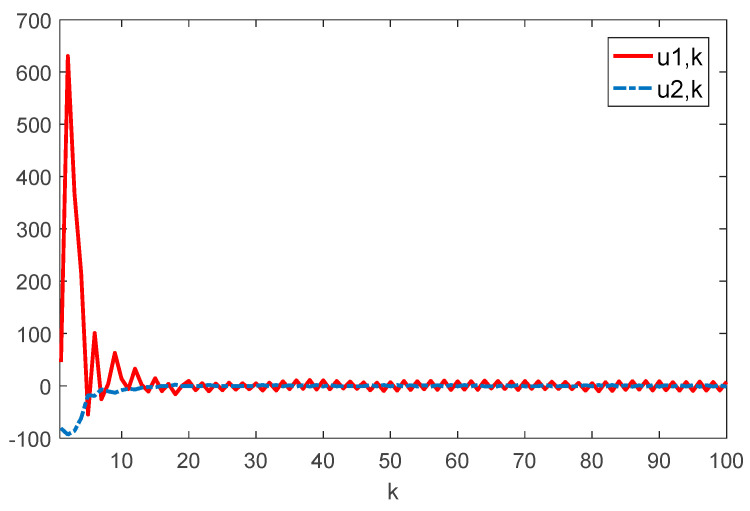
Time evolution of $u_{i\cdot k}(i=1,2)$ with controller (17).

Figure 6, Figure 7 and Figure 8 show that the controller designed in this paper can keep the system in a stable state under different packet loss rates.

**Remark** **2.** *External disturbance and time delays are not considered in references [29,32], respectively. Meanwhile, the references [29,30,32] do not consider stochastic uncertainty, and references [29,30] ignore the two-channel packet dropouts. In Table 1, the model of this paper contains more influencing factors, and it is more general than references [29,30,32].*

## 7. Conclusions

In this paper, the robust H_∞_ SMC stability problem has been discussed for uncertain time-varying delays systems with stochastic uncertainty, external disturbance, and two-channel packet dropouts. In order to suppress the effects of packet dropouts on system stability, a robust sliding surface was applied, which proved to be more robust against two-channel packet dropouts, stochastic uncertainty, external disturbances, and time delays. Then, sufficient conditions have been gained to ensure the robust mean-square asymptotic stability of the sliding mode dynamics with the H_∞_ performance. Furthermore, a sliding mode controller with an uncertainty compensation term is proposed, which can inhibit the influence of uncertainties on systems. Finally, two numerical examples have been given to demonstrate the feasibility of the obtained H_∞_ SMC scheme. In the future, we will investigate Markov jump systems with two channels—the sensor–controller channel, and the controller–actuator channel packet dropouts—and the non-fragile control of two-channel packet dropouts. We can also study the problem of robust fault detection in networked control systems. Considering the packet dropout compensation, when there are time delays, two-channel packet dropouts, and quantization errors, we can construct corresponding fault detection filters to detect the system faults as well as more practical controllers to discuss more complex systems.

## Figures and Tables

**Table 1:** Model comparisons.

Model	This Paper	[29]	[30]	[32]
Time delays	√	√	√	×
Stochastic uncertainty	√	×	×	×
External disturbance	√	×	√	√
Two-channel packet dropouts	√	×	×	√

Where “√” means that the model contains this component, and “×” means that the model does not contain this component.

## Data Availability

Not applicable.

## Nomenclature

The relevant notations employed are standard, P,Q,R,Λ are the symmetric matrices required by this paper, respectively. ε is the positive number to be calculated in this paper. Some other details are shown as follows.

$R^{n}$	The n-dimensional Euclidean space
$M^{T}$/$M^{-1}$	The transposition/inverse of matrix M
*	The entries caused by symmetry
Pr{⋅}	The occurrence probability of the event ‘·’
E{x}	The expectation of x
‖⋅‖	The Euclidean norm
X>0	The matrix X is positive definite
diag{⋯}	The block diagonal matrix
I	The identity matrix with compatible dimension

## Appendix A. Proof of Theorem 1

The Lyapunov–Krasovskii functional is designed as follows:

(A1) $$V_{k}=\sum_{i=1}^{3}V_{i\cdot k},$$

where

$V_{1\cdot k}=x_{k}^{T}Px_{k}+x_{k-1}^{T}Rx_{k-1}+u_{k-1}^{T}\Lambda u_{k-1}$,$V_{2\cdot k}=\sum_{j=k-d_{k}}^{k-1}x_{j}^{T}Qx_{j}$,$V_{3\cdot k}=\sum_{j=-d_{M}+1}^{-d_{m}}\sum_{i=k+j}^{k-1}x_{i}^{T}Qx_{i}$, with P>0,R>0,Λ>0 and Q>0 are matrices to be determined. Then, along the trajectory of system (9), we have

(A2) $$E\{\Delta V_{k}\}=\sum_{i=1}^{3}E\{\Delta V_{i\cdot k}\},$$

where

(A3) $$\begin{array}{c} E\{\Delta V_{1\cdot k}\}\\ =E\{x_{k+1}^{T}Px_{k+1}+x_{k}^{T}Rx_{k}+u_{k}^{T}\Lambda u_{k}-x_{k}^{T}Px_{k}-x_{k-1}^{T}Rx_{k-1}-u_{k-1}^{T}\Lambda u_{k-1}\}\\ =E\{x_{k}^{T}{\left(\tilde{A}-\Delta \bar{A}\right)}^{T}P(\tilde{A}-\Delta \bar{A})x_{k}+2x_{k}^{T}{\left(\tilde{A}-\Delta \bar{A}\right)}^{T}P({\tilde{A}}_{d}-\Delta {\bar{A}}_{d})x_{k-d_{k}}\\ -2\lambda_{1}x_{k}^{T}{\left(\tilde{A}-\Delta \bar{A}\right)}^{T}P\bar{A}x_{k-1}-2\lambda_{2}x_{k}^{T}{\left(\tilde{A}-\Delta \bar{A}\right)}^{T}PBu_{k-1}\\ +x_{k-d_{k}}^{T}{\left({\tilde{A}}_{d}-\Delta {\bar{A}}_{d}\right)}^{T}P({\tilde{A}}_{d}-\Delta {\bar{A}}_{d})x_{k-d_{k}}-2\lambda_{1}x_{k-d_{k}}^{T}{\left({\tilde{A}}_{d}-\Delta {\bar{A}}_{d}\right)}^{T}P\bar{A}x_{k-1}\\ -2\lambda_{2}x_{k-d_{k}}^{T}{\left({\tilde{A}}_{d}-\Delta {\bar{A}}_{d}\right)}^{T}PBu_{k-1}+\lambda_{1}^{2}x_{k-1}^{T}{\bar{A}}^{T}P\bar{A}x_{k-1}\\ +2\lambda_{1}\lambda_{2}x_{k-1}^{T}{\bar{A}}^{T}PBu_{k-1}+\lambda_{2}^{2}u_{k-1}^{T}B^{T}PBu_{k-1}+\bar{\alpha }x_{k}^{T}\Delta A^{T}P\Delta Ax_{k}\\ +\bar{\beta }x_{k-d_{k}}^{T}\Delta A_{d}^{T}P\Delta A_{d}x_{k-d_{k}}+x_{k}^{T}{\tilde{A}}^{T}G^{T}\bar{\Lambda }G\tilde{A}x_{k}+2x_{k}^{T}{\tilde{A}}^{T}G^{T}\bar{\Lambda }G{\tilde{A}}_{d}x_{k-d_{k}}\\ +2\lambda_{1}x_{k}^{T}{\tilde{A}}^{T}G^{T}\bar{\Lambda }GAx_{k-1}+2\lambda_{2}x_{k}^{T}{\tilde{A}}^{T}{\tilde{G}}^{T}\Lambda u_{k-1}+x_{k-d_{k}}^{T}{\tilde{A}}_{d}{}^{T}G^{T}\bar{\Lambda }G{\tilde{A}}_{d}x_{k-d_{k}}\\ +2\lambda_{1}x_{k-d_{k}}^{T}{\tilde{A}}_{d}{}^{T}G^{T}\bar{\Lambda }GAx_{k-1}+2\lambda_{2}x_{k-d_{k}}^{T}{\tilde{A}}_{d}{}^{T}{\tilde{G}}^{T}\Lambda u_{k-1}\\ +\lambda_{1}^{2}x_{k-1}^{T}A^{T}G^{T}\bar{\Lambda }GAx_{k-1}+2\lambda_{1}\lambda_{2}x_{k-1}^{T}A^{T}{\tilde{G}}^{T}\Lambda u_{k-1}\\ +\lambda_{2}^{2}u_{k-1}^{T}\Lambda u_{k-1}+x_{k}^{T}Rx_{k}-x_{k}^{T}Px_{k}-x_{k-1}^{T}Rx_{k-1}-u_{k-1}^{T}\Lambda u_{k-1}\}, \end{array}$$

where $\tilde{A}=A+\alpha \Delta A$, ${\tilde{A}}_{d}=A_{d}+\beta \Delta A_{d}$.

By using the elementary matrix inequality technique, it follows that

(A4) $$\begin{array}{l} 2x_{k}^{T}{\left(\tilde{A}-\Delta \bar{A}\right)}^{T}P({\tilde{A}}_{d}-\Delta {\bar{A}}_{d})x_{k-d_{k}}\\ \leq x_{k}^{T}{\left(\tilde{A}-\Delta \bar{A}\right)}^{T}P(\tilde{A}-\Delta \bar{A})x_{k}+x_{k-d_{k}}^{T}{\left({\tilde{A}}_{d}-\Delta {\bar{A}}_{d}\right)}^{T}P({\tilde{A}}_{d}-\Delta {\bar{A}}_{d})x_{k-d_{k}}\\ \leq 2x_{k}^{T}{\tilde{A}}^{T}P\tilde{A}x_{k}+2x_{k}^{T}{\tilde{A}}^{T}G^{T}{\left(GB\right)}^{-1}G\tilde{A}x_{k}+2x_{k-d_{k}}^{T}{\tilde{A}}_{d}{}^{T}P{\tilde{A}}_{d}x_{k-d_{k}}\\ +2x_{k-d_{k}}^{T}{\tilde{A}}_{d}{}^{T}G^{T}{\left(GB\right)}^{-1}G{\tilde{A}}_{d}x_{k-d_{k}}, \end{array}$$

and by applying Lemma 1, it follows that

(A5) $$\begin{array}{l} -2\lambda_{1}x_{k}^{T}{\left(\tilde{A}-\Delta \bar{A}\right)}^{T}P\bar{A}x_{k-1}\\ \leq \lambda_{1}x_{k}^{T}{\left(\tilde{A}-\Delta \bar{A}\right)}^{T}P(\tilde{A}-\Delta \bar{A})x_{k}+\lambda_{1}x_{k-1}^{T}{\bar{A}}^{T}P\bar{A}x_{k-1}\\ \leq 2\lambda_{1}x_{k}^{T}{\tilde{A}}^{T}P\tilde{A}x_{k}+2\lambda_{1}x_{k}^{T}{\tilde{A}}^{T}G^{T}{\left(GB\right)}^{-1}G\tilde{A}x_{k}+\lambda_{1}x_{k-1}^{T}{\bar{A}}^{T}P\bar{A}x_{k-1}. \end{array}$$

The other terms are deduced in the same way. Hence, substituting Equations (A4) and (A5) into (A3) yields

(A6) $$\begin{array}{c} E\{\Delta V_{1\cdot k}\}\\ =E\{2\xi x_{k}^{T}{\tilde{A}}^{T}P\tilde{A}x_{k}+2\xi x_{k}^{T}{\tilde{A}}^{T}G^{T}{\left(GB\right)}^{-1}G\tilde{A}x_{k}+\bar{\alpha }x_{k}^{T}\Delta A^{T}P\Delta Ax_{k}\\ +\xi x_{k}^{T}{\tilde{A}}^{T}G^{T}\bar{\Lambda }G\tilde{A}x_{k}+2\xi x_{k-d_{k}}^{T}{\tilde{A}}_{d}{}^{T}P{\tilde{A}}_{d}x_{k-d_{k}}+x_{k}^{T}Rx_{k}-x_{k}^{T}Px_{k}\\ +2\xi x_{k-d_{k}}^{T}{\tilde{A}}_{d}{}^{T}G^{T}{\left(GB\right)}^{-1}G{\tilde{A}}_{d}x_{k-d_{k}}+\bar{\beta }x_{k-d_{k}}^{T}\Delta A_{d}^{T}P\Delta A_{d}x_{k-d_{k}}\\ +\xi x_{k-d_{k}}^{T}{\tilde{A}}_{d}{}^{T}G^{T}\bar{\Lambda }G{\tilde{A}}_{d}x_{k-d_{k}}+gx_{k-1}^{T}{\bar{A}}^{T}P\bar{A}x_{k-1}-x_{k-1}^{T}Rx_{k-1}\\ +gx_{k-1}^{T}A^{T}G^{T}\bar{\Lambda }GAx_{k-1}+hu_{k-1}^{T}B^{T}PBu_{k-1}+hu_{k-1}^{T}\Lambda u_{k-1}-u_{k-1}^{T}\Lambda u_{k-1}\}. \end{array}$$

Similarly, it can be inferred that

(A7) $$E\{\Delta V_{2\cdot k}\}\leq E\left\{x_{k}^{T}Qx_{k}-x_{k-d_{k}}^{T}Qx_{k-d_{k}}+\sum_{j=k-d_{M}+1}^{k-d_{m}}x_{j}^{T}Qx_{j}\right\}.$$

(A8) $$E\{\Delta V_{3\cdot k}\}=E\left\{(d_{M}-d_{m})x_{k}^{T}Qx_{k}-\sum_{j=k-d_{M}+1}^{k-d_{m}}x_{j}^{T}Qx_{j}\right\}.$$

Thus,

(A9) $$\begin{array}{l} E\{\Delta V_{k}\}\\ \leq E\{2\xi x_{k}^{T}{\tilde{A}}^{T}P\tilde{A}x_{k}+2\xi x_{k}^{T}{\tilde{A}}^{T}G^{T}{\left(GB\right)}^{-1}G\tilde{A}x_{k}+\bar{\alpha }x_{k}^{T}\Delta A^{T}P\Delta Ax_{k}\\ +\xi x_{k}^{T}{\tilde{A}}^{T}G^{T}\bar{\Lambda }G\tilde{A}x_{k}+x_{k}^{T}Rx_{k}-x_{k}^{T}Px_{k}+(d_{M}-d_{m}+1)x_{k}^{T}Qx_{k}\\ -x_{k-d_{k}}^{T}Qx_{k-d_{k}}+2\xi x_{k-d_{k}}^{T}{\tilde{A}}_{d}{}^{T}P{\tilde{A}}_{d}x_{k-d_{k}}+2\xi x_{k-d_{k}}^{T}{\tilde{A}}_{d}{}^{T}G^{T}{\left(GB\right)}^{-1}G{\tilde{A}}_{d}x_{k-d_{k}}\\ +\bar{\beta }x_{k-d_{k}}^{T}\Delta A_{d}^{T}P\Delta A_{d}x_{k-d_{k}}+\xi x_{k-d_{k}}^{T}{\tilde{A}}_{d}{}^{T}G^{T}\bar{\Lambda }G{\tilde{A}}_{d}x_{k-d_{k}}\\ +gx_{k-1}^{T}{\bar{A}}^{T}P\bar{A}x_{k-1}+gx_{k-1}^{T}A^{T}G^{T}\bar{\Lambda }GAx_{k-1}+hu_{k-1}^{T}B^{T}PBu_{k-1}\\ +hu_{k-1}^{T}\Lambda u_{k-1}-x_{k-1}^{T}Rx_{k-1}-u_{k-1}^{T}\Lambda u_{k-1}\}=E\{\eta_{k}^{T}\Omega \eta_{K}\}<0, \end{array}$$

where

$$\eta_{k}={\left[\begin{array}{cccc} x_{k}^{T} & x_{k-d_{k}}^{T} & x_{k-1}^{T} & u_{k-1}^{T} \end{array}\right]}^{T},$$

$$\Omega =diag\{\Omega_{11},\Omega_{22},\Omega_{33},\Omega_{44}\},$$

$$\begin{array}{cc} \Omega_{11} & =2\xi {\tilde{A}}^{T}P\tilde{A}+2\xi {\tilde{A}}^{T}G^{T}{\left(GB\right)}^{-1}G\tilde{A}+\bar{\alpha }\Delta A^{T}P\Delta A+\xi {\tilde{A}}^{T}G^{T}\bar{\Lambda }G\tilde{A}-P+R\\ & +(d_{M}-d_{m}+1)Q, \end{array}$$

$$\Omega_{22}=2\xi {\tilde{A}}^{T}P\tilde{A}+2\xi {\tilde{A}}^{T}G^{T}{\left(GB\right)}^{-1}G\tilde{A}+\bar{\beta }\Delta A_{d}^{T}P\Delta A_{d}+\xi {\tilde{A}}^{T}G^{T}\bar{\Lambda }G\tilde{A}-Q,$$

$$\Omega_{33}=g{\bar{A}}^{T}P\bar{A}+gA^{T}G^{T}\bar{\Lambda }GA-R,$$

$$\Omega_{44}=hB^{T}PB+h\Lambda -\Lambda .$$

Subsequently, noting $G=B^{T}P$ and employing Lemma 2, Ω<0 is equivalent to

(A10) $$\prod =\left[\begin{array}{ccc} \prod_{11} & \prod_{12} & \prod_{13}\\ {}* & \prod_{22} & 0\\ {}* & * & \prod_{33} \end{array}\right]<0,$$

where

$$\prod_{11}=\left[\begin{array}{ccccc} -P+R+(d_{M}-d_{m}+1)Q & 0 & 0 & \sqrt{2\xi }{\tilde{A}}^{T}P & \sqrt{2\xi }{\tilde{A}}^{T}PB\\ {}* & -Q & 0 & 0 & 0\\ {}* & * & -R & 0 & 0\\ {}* & * & * & -P & 0\\ {}* & * & * & * & -B^{T}PB \end{array}\right],$$

$$\prod_{12}=[\begin{array}{cc} {\bar{\prod }}_{12} & {\prod^{⌢}}_{12} \end{array}],{\bar{\prod }}_{12}=\left[\begin{array}{cc} \sqrt{\bar{\alpha }}\Delta A^{T}P & \sqrt{\xi }{\tilde{A}}^{T}PB\\ 0_{4\times 1} & 0_{4\times 1} \end{array}\right],{\prod^{⌢}}_{12}=\left[\begin{array}{c} 0_{1\times 3}\\ \prod_{12}^{*}\\ 0_{3\times 3} \end{array}\right],$$

$$\prod_{12}^{*}=[\begin{array}{ccc} \sqrt{2\xi }{\tilde{A}}_{d}{}^{T}P & \sqrt{2\xi }{\tilde{A}}_{d}{}^{T}PB & \sqrt{\bar{\beta }}\Delta A_{d}{}^{T}P \end{array}],$$

$$\prod_{22}=diag\{-P,-{\bar{\Lambda }}^{-1},-P,-B^{T}PB,-P\},\prod_{33}=diag\{-{\bar{\Lambda }}^{-1},-B^{T}PB,-{\bar{\Lambda }}^{-1},-P,-{\bar{\Lambda }}^{-1}\},$$

$$\prod_{13}=\left[\begin{array}{ccccc} 0 & 0 & 0 & 0 & 0\\ \sqrt{\xi }{\tilde{A}}_{d}{}^{T}PB & 0 & 0 & 0 & 0\\ 0 & \sqrt{g}A^{T}PB & \sqrt{g}A^{T}PB & 0 & 0\\ 0 & 0 & 0 & 0 & 0\\ 0 & 0 & 0 & \sqrt{h}B^{T}P & \sqrt{1-h}B^{T}PB \end{array}\right].$$

By using the Schur complement, rewrite matrix ∏ by

(A11) $$\prod =\tilde{\prod }+\hat{H}\hat{F}\hat{N}+{\hat{N}}^{T}{\hat{F}}^{T}{\hat{H}}^{T},$$

where

$$\tilde{\prod }=\left[\begin{array}{ccc} {\tilde{\prod }}_{11} & {\tilde{\prod }}_{12} & {\tilde{\prod }}_{13}\\ {}* & \prod_{22} & 0\\ {}* & * & \prod_{33} \end{array}\right],{\tilde{\prod }}_{12}=\left[\begin{array}{c} \prod_{1}\\ \prod_{2}\\ 0_{3\times 5} \end{array}\right],\hat{H}=\left[\begin{array}{cc} {\hat{H}}_{11} & 0\\ 0 & {\hat{H}}_{22} \end{array}\right],\hat{N}=\left[\begin{array}{ccc} \varepsilon N^{T} & 0 & 0_{1\times 13}\\ 0 & \varepsilon N^{T} & 0_{1\times 13} \end{array}\right],$$

$${\tilde{\prod }}_{11}=\left[\begin{array}{ccccc} -P+R+(d_{M}-d_{m}+1)Q & 0 & 0 & \sqrt{2\xi }A^{T}P & \sqrt{2\xi }A^{T}PB\\ {}* & -Q & 0 & 0 & 0\\ {}* & * & -R & 0 & 0\\ {}* & * & * & -P & 0\\ {}* & * & * & * & -B^{T}PB \end{array}\right],$$

$$\prod_{1}=[\begin{array}{ccccc} 0 & \sqrt{\xi }A^{T}PB & 0 & 0 & 0 \end{array}],\prod_{2}=[\begin{array}{ccccc} 0 & 0 & \sqrt{2\xi }A_{d}{}^{T}P & \sqrt{2\xi }A_{d}{}^{T}PB & 0 \end{array}],$$

$${\tilde{\prod }}_{13}=\left[\begin{array}{ccccc} 0 & 0 & 0 & 0 & 0\\ \sqrt{\xi }A_{d}{}^{T}PB & 0 & 0 & 0 & 0\\ 0 & \sqrt{g}A^{T}PB & \sqrt{g}A^{T}PB & 0 & 0\\ 0 & 0 & 0 & 0 & 0\\ 0 & 0 & 0 & \sqrt{h}B^{T}P & \sqrt{1-h}B^{T}PB \end{array}\right],$$

$${\hat{H}}_{11}={\left[\begin{array}{ccccccc} 0 & 0 & 0 & \sqrt{2\xi }\alpha PH & \sqrt{2\xi }\alpha B^{T}PH & \sqrt{\bar{\alpha }}PH & \sqrt{\xi }\alpha B^{T}PH \end{array}\right]}^{T},$$

$${\hat{H}}_{22}={\left[\begin{array}{cccccccc} \sqrt{2\xi }\beta PH_{d} & \sqrt{2\xi }\beta B^{T}PH_{d} & \sqrt{\bar{\beta }}PH_{d} & \sqrt{\xi }\beta B^{T}PH_{d} & 0 & 0 & 0 & 0 \end{array}\right]}^{T}.$$

By using Lemmas 2 and 3, it is obtained that Ψ<0 can guarantee matrix ∏<0; then, we can guarantee matrix Ω<0; then, $E\{\Delta V_{k}\}<0$. Therefore, it can be concluded that the sliding mode dynamics (9) are robustly asymptotically stable in a mean square sense. Then, the proof of Theorem 1 is complete.

## Appendix B. Proof of Theorem 2

We will handle the H_∞_ performance of the dynamics. The same Lyapunov–Krasovskii functional is chosen as in Theorem 1. By a similar approach, we can obtain the following inequality,

(A12) $$E\{\Delta V_{k}\}\leq E\{{\tilde{\eta }}_{k}^{T}\Theta {\tilde{\eta }}_{k}\},$$

where

$${\tilde{\eta }}_{k}={\left[\begin{array}{cc} \eta_{k}^{T} & \omega_{k}^{T} \end{array}\right]}^{T},$$

$$\Theta =diag\{\Theta_{11},\Theta_{22},\Theta_{33},\Theta_{44},\Theta_{55}\},$$

$$\begin{array}{cc} \Theta_{11} & =2\bar{\xi }{\tilde{A}}^{T}P\tilde{A}+2\bar{\xi }{\tilde{A}}^{T}G^{T}{\left(GB\right)}^{-1}G\tilde{A}+\bar{\xi }{\tilde{A}}^{T}G^{T}\bar{\Lambda }G\tilde{A}+\bar{\alpha }\Delta A^{T}P\Delta A-P+R\\ & +(d_{M}-d_{m}+1)Q, \end{array}$$

$$\Theta_{22}=2\bar{\xi }{\tilde{A}}_{d}{}^{T}P{\tilde{A}}_{d}+2\bar{\xi }{\tilde{A}}_{d}{}^{T}G^{T}{\left(GB\right)}^{-1}G{\tilde{A}}_{d}+\bar{\xi }{\tilde{A}}_{d}{}^{T}G^{T}\bar{\Lambda }G{\tilde{A}}_{d}+\bar{\beta }\Delta A_{d}{}^{T}P\Delta A_{d}-Q,$$

$$\Theta_{33}=\bar{g}A^{T}G^{T}\bar{\Lambda }GA+\bar{g}A^{T}G^{T}{\left(GB\right)}^{-1}GA-R,\Theta_{44}=\bar{h}\Lambda +\bar{h}B^{T}PB-\Lambda ,$$

$$\Theta_{55}=2\bar{\xi }D^{T}PD+2\bar{\xi }D^{T}G^{T}{\left(GB\right)}^{-1}GD+\bar{\xi }D^{T}G^{T}\bar{\Lambda }GD.$$

The following index is introduced to deal with the H_∞_ performance analyses of the sliding mode dynamics (9), assuming $\phi_{k}=0$, which leads to

(A13) $$\begin{array}{cc} \Gamma_{n} & =E\{\sum_{k=0}^{n}\left[z_{k}^{T}z_{k}-\gamma^{2}\omega_{k}^{T}\omega_{k}\right]\}\\ & =E\{\sum_{k=0}^{n}\left[z_{k}^{T}z_{k}-\gamma^{2}\omega_{k}^{T}\omega_{k}+\Delta V_{k}\right]-V_{n-1}\}\\ & \leq E\{{\tilde{\eta }}_{k}^{T}\bar{\Theta }\tilde{\eta }\}, \end{array}$$

where

$$\bar{\Theta }=\left[\begin{array}{ccccc} {\bar{\Theta }}_{11} & 0 & 0 & 0 & {\bar{\Theta }}_{15}\\ {}* & {\bar{\Theta }}_{22} & 0 & 0 & 0\\ {}* & * & {\bar{\Theta }}_{33} & 0 & 0\\ {}* & * & * & {\bar{\Theta }}_{44} & 0\\ {}* & * & * & * & {\bar{\Theta }}_{55} \end{array}\right],$$

$$\begin{array}{cc} {\bar{\Theta }}_{11} & =2\bar{\xi }{\tilde{A}}^{T}P\tilde{A}+2\bar{\xi }{\tilde{A}}^{T}G^{T}{\left(GB\right)}^{-1}G\tilde{A}+\bar{\alpha }\Delta A^{T}P\Delta A+\bar{\xi }{\tilde{A}}^{T}G^{T}\bar{\Lambda }G\tilde{A}-P+R+C^{T}C\\ & +(d_{M}-d_{m}+1)Q, \end{array}$$

$${\bar{\Theta }}_{22}=2\bar{\xi }{\tilde{A}}_{d}{}^{T}P{\tilde{A}}_{d}+2\bar{\xi }{\tilde{A}}_{d}{}^{T}G^{T}{\left(GB\right)}^{-1}G{\tilde{A}}_{d}+\bar{\xi }{\tilde{A}}_{d}{}^{T}G^{T}\bar{\Lambda }G{\tilde{A}}_{d}+\bar{\beta }\Delta A_{d}{}^{T}P\Delta A_{d}-Q,$$

$${\bar{\Theta }}_{33}=\bar{g}A^{T}G^{T}\bar{\Lambda }GA+\bar{g}A^{T}G^{T}{\left(GB\right)}^{-1}GA-R,{\bar{\Theta }}_{44}=\bar{h}\Lambda +\bar{h}B^{T}PB-\Lambda ,$$

$${\bar{\Theta }}_{55}=2\bar{\xi }D^{T}PD+2\bar{\xi }D^{T}G^{T}{\left(GB\right)}^{-1}GD+\bar{\xi }D^{T}G^{T}\bar{\Lambda }GD+F^{T}F-\gamma^{2}Ι,{\bar{\Theta }}_{15}=C^{T}F.$$

We can obtain that $\bar{\Theta }<0$ is equivalent to (14), and it yields $\Gamma_{n}<0$, which results in ${\left\|z_{k}\right\|}_{2}^{2}<\gamma^{2}{\left\|\omega_{k}\right\|}_{2}^{2}$, completing the proof of Theorem 2.

## References

[1] Tipsuwan Y., Chow M.Y. (2003). Control methodologies in networked control systems. Control Eng. Pract..

[2] Zhang J., Xia Y., Shi P. (2012). Design and stability analysis of networked predictive control systems. IEEE Trans. Control Syst. Technol..

[3] Sun K., Qiu J., Karimi H.R. (2019). A novel finite-time control for nonstrict feedback saturated nonlinear systems with tracking error constraint. IEEE Trans. Syst. Man Cybern. Syst..

[4] Kim G.H., Hong K.S. (2019). Adaptive sliding-mode control of an offshore container crane with unknown disturbances. IEEE/ASME Trans. Mechatron..

[5] Xu J.X., Guo Z.Q., Lee T.H. (2013). Design and implementation of integral sliding-mode control on an underactuated two-wheeled mobile robot. IEEE Trans. Ind. Electron..

[6] Tong D.B., Xu C., Chen Q.Y. (2020). Sliding mode control for nonlinear stochastic systems with Markovian jumping parameters and mode-dependent time-varying delays. Nonlinear Dyn..

[7] Xia Y., Jia Y. (2003). Robust sliding-mode control for uncertain time-delay systems: An LMI approach. IEEE Trans. Autom. Control.

[8] Zhang J., Lyu M., Shen T. (2017). Sliding mode control for a class of nonlinear multi-agent system with time delay and uncertainties. IEEE Trans. Ind. Electron..

[9] Su L., Chesi G. (2018). Robust stability of uncertain linear systems with input and output quantization and packet loss. Automatica.

[10] Hu J., Zhang H., Yu X. (2019). Design of sliding-mode-based control for nonlinear systems with mixed-delays and packet losses under uncertain missing probability. IEEE Trans. Syst. Man Cybern. Syst..

[11] Li J., Niu Y., Song J. (2021). Sliding mode control design under multiple nodes round-robin-like protocol and packet length-dependent lossy network. Automatica.

[12] Ding J., Sun S., Ma J. (2019). Fusion estimation for multi-sensor networked systems with packet loss compensation. Inf. Fusion.

[13] Hentati A., Frigon J.F., Ajib W. (2019). Energy harvesting wireless sensor networks with channel estimation: Delay and packet loss performance analysis. IEEE Trans. Veh. Technol..

[14] Pradhan S.K., Das D.K. (2020). H∞ Load frequency control design based on delay discretization approach for interconnected power systems with time delay. J. Mod. Power Syst. Clean Energy.

[15] Cai X., Zhong S., Wang J. (2020). Robust H_∞_ control for uncertain delayed TS fuzzy systems with stochastic packet dropouts. Appl. Math. Comput..

[16] Jiang X., Han Q.L., Liu S. (2008). A New H_∞_ Stabilization Criterion for Networked Control Systems. IEEE Trans. Autom. Control.

[17] Lu R., Xu Y., Zhang R. (2016). A new design of model predictive tracking control for networked control system under random packet loss and uncertainties. IEEE Trans. Ind. Electron..

[18] Shah D., Mehta A. (2017). Discrete-time sliding mode controller subject to real-time fractional delays and packet losses for networked control system. Int. J. Control Autom. Syst..

[19] Chang X.H., Huang R., Wang H. (2019). Robust design strategy of quantized feedback control. IEEE Trans. Circuits Syst. II Express Briefs.

[20] Shen Y., Wu Z.G., Shi P. (2019). H_∞_ control of Markov jump time-delay systems under asynchronous controller and quantizer. Automatica.

[21] Guo Y., Hou Z., Liu S. (2019). Data-driven model-free adaptive predictive control for a class of MIMO nonlinear discrete-time systems with stability analysis. IEEE Access.

[22] Cassandras C.G., Pepyne D.L., Wardi Y. (2001). Optimal control of a class of hybrid systems. IEEE Trans. Autom. Control.

[23] Xu C., Tong D.B., Chen Q.Y. (2021). Exponential stability of Markovian jumping systems via adaptive sliding mode control. IEEE Trans. Syst. Man Cybern. Syst..

[24] Wang J., Yang C., Shen H. (2020). Sliding-mode control for slow-sampling singularly perturbed systems subject to Markov jump parameters. IEEE Trans. Syst. Man Cybern. Syst..

[25] Roy S., Baldi S., Fridman L.M. (2020). On adaptive sliding mode control without a priori bounded uncertainty. Automatica.

[26] Wang S., Tao L., Chen Q. (2020). USDE-based sliding mode control for servo mechanisms with unknown system dynamics. IEEE/ASME Trans. Mechatron..

[27] Zhan X.S., Wu J., Jiang T. (2015). Optimal performance of networked control systems under the packet dropouts and channel noise. ISA Trans..

[28] Niu Y., Ho D.W. (2010). Design of sliding mode control subject to packet losses. IEEE Trans. Autom. Control.

[29] Zhang P., Hu J., Zhang H. Robust sliding mode control for discrete delayed systems with randomly varying nonlinearities under uncertain occurrence probability. Proceedings of the 2017 Chinese Automation Congress (CAC).

[30] Zhang P., Hu J., Zhang H. (2018). Robust H∞ control for delayed systems with randomly varying nonlinearities under uncertain occurrence probability via sliding mode method. Syst. Sci. Control Eng..

[31] Zhang D., Zhang Y. (2020). Fault Detection for uncertain delta operator systems with two-channel packet dropouts via a switched systems approach. J. Syst. Sci. Complex..

[32] Zhang Y., Ren L., Xie S. (2019). Robust sliding mode control for uncertain networked control system with two-channel packet dropouts. J. Cent. South Univ..

[33] Chen H., Gao J., Shi T. (2016). H∞ control for networked control systems with time delay, data packet dropout and disorder. Neurocomputing.

[34] Kasana R., Kumar S., Kaiwartya O. (2018). Fuzzy-based channel selection for location oriented services in multichannel VCPS environments. IEEE Internet Things J..

[35] Makarfi A.U., Rabie K.M., Kaiwartya O. Reconfigurable intelligent surface enabled IoT networks in generalized fading channels. Proceedings of the ICC 2020—2020 IEEE International Conference on Communications (ICC).

[36] Bahreini M., Zarei J. (2020). Robust finite-time fault-tolerant control for networked control systems with random delays: A Markovian jump system approach. Nonlinear Anal. Hybrid Syst..

[37] Li M., Chen Y. (2018). Robust time-varying H∞ control for networked control system with uncertainties and external disturbance. Int. J. Control Autom. Syst..

[38] Wang C., Li R., Su X. (2021). Output Feedback Sliding Mode Control of Markovian Jump Systems and Its Application to Switched Boost Converter. IEEE Trans. Circuits Syst. I Regul. Pap..

[39] Zhao H., Niu Y., Zhao J. (2019). Event-triggered sliding mode control of uncertain switched systems under denial-of-service attacks. J. Frankl. Inst..

[40] Wei Y., Park J.H., Qiu J. (2017). Sliding mode control for semi-Markovian jump systems via output feedback. Automatica.

[41] Ban J., Seo M., Goh T. (2020). Improved co-design of event-triggered dynamic output feedback controllers for linear systems. Automatica.

[42] Wang J.L., Wu H.N., Huang T. (2015). Pinning control for synchronization of coupled reaction-diffusion neural networks with directed topologies. IEEE Trans. Syst. Man Cybern. Syst..

[43] Boyd S., El G.L., Feron E. (1994). Linear Matrix Inequalities in System and Control Theory.

[44] Hu J., Wang Z., Gao H. (2012). Robust H_∞_ sliding mode control for discrete time-delay systems with stochastic nonlinearities. J. Frankl. Inst..

